# Editorial: Molecular Mechanisms of Heritable Connective Tissue Disorders

**DOI:** 10.3389/fgene.2022.866665

**Published:** 2022-04-27

**Authors:** Fransiska Malfait, Antonella Forlino, Gerhard Sengle, Tom Van Agtmael

**Affiliations:** ^1^ Centre for Medical Genetics, Ghent University Hospital, Ghent, Belgium; ^2^ Department of Biomolecular Medicine, Ghent University, Ghent, Belgium; ^3^ Department of Molecular Medicine, Biochemistry Unit, University of Pavia, Pavia, Italy; ^4^ Center for Biochemistry, Faculty of Medicine, University Hospital of Cologne, Cologne, Germany; ^5^ Department of Pediatrics and Adolescent Medicine, Faculty of Medicine and University Hospital Cologne, University of Cologne, Cologne, Germany; ^6^ Center for Molecular Medicine Cologne (CMMC), University of Cologne, Cologne, Germany; ^7^ Cologne Center for Musculoskeletal Biomechanics (CCMB), Cologne, Germany; ^8^ Institute of Cardiovascular and Medical Sciences, College of Medical, Veterinary & Life Sciences, University of Glasgow, Glasgow, United Kingdom

**Keywords:** extracellular matrix, heritable disorders of connective tissue, connective tissue, collagen, proteoglycans (PG), fibrillin (FBN)

Tissue microenvironments formed by extracellular matrix (ECM) networks play a crucial role in regulating tissue structure and function. Extracellular microfibrillar networks composed of large multidomain glycoproteins such as collagens and fibrillins are of particular interest in this regard since they surround cells in close proximity to basement membranes, and thereby guide proper morphology and functional behavior of specialized cell types. Genetic defects in essential building blocks of the ECM architecture lead to multi-system connective tissue disorders, for instance affecting the musculoskeletal, dermal, vascular, ocular, and renal system, collectively called heritable connective tissue disorders (HCTD). Tissue weakness caused by ECM protein defects often correlates with dysregulated growth and differentiation processes due to an abnormal growth factor bioavailability.

Due to the striking clinical features of patients affected by HCTD, investigating the underlying molecular mechanisms allows to reveal fundamental pathways in embryonic development and postnatal tissue homeostasis. This research is not only required for the development of new therapeutic avenues for affected individuals, but also to tackle challenges of general medicine in age-related diseases accompanied by connective tissue degeneration and failed remodelling, and increases our insight into fundamental molecular mechanisms of cell and tissue function.

This Research Topic issue highlights molecular pathways underlying HCTD. Supramolecular networks consisting of collagen and elastic fibers not only provide tissue integrity to connective tissues, but also define their biomechanical properties. This mutual interdependence is also required for the proper control of ECM-cell communication that is facilitated by specific ECM receptors or growth factors. The structural and functional interdependence of both major networks is reflected by common clinical features of patients with deficiencies in collagen or elastic fiber network components, that are often accompanied by similar dysregulated signaling pathways. For instance, aberrant TGF-β and integrin signaling have been shown to be involved in several types of osteogenesis imperfecta (OI) (Grafe et al., 2014; Etich et al., 2020) epidermolysis bullosa (EB) (Kiritsi and Nystrom, 2018; Tartaglia et al., 2021), Ehlers-Danlos (EDS) (Morissette et al., 2014), cutis laxa (CL) (Urban and Davis, 2014), and in the fibrillinopathies (Sengle and Sakai, 2015).

Cardinal et al. report on a therapeutic intervention for osteogenesis imperfecta (OI), a disorder characterized by brittle bones, often due to a collagen type I gene mutation, in a preclinical murine model (oim/oim) by anti-sclerostin antibody administration.

Inherited disorders characterized by deficiencies in basement membrane structure and function caused by laminin mutations are the focus of a review by Shaw et al., with a particular emphasis on the lessons that can be learned from analysing laminin mutations in terms of laminin biology in health and disease. Nyström et al. focus on the basement membrane protein collagen VII and its role in dystrophic epidermolysis bullosa, a skin blistering disease that is caused by mutations in *COL7A1*. The skin features of patients affected by dystrophic epidermolysis bullosa vary from mild blistering primarily affecting hands, feet, knees, and elbows to severe cases with widespread blistering that can lead to vision loss, and scarring. Nyström et al. cover genetic modifiers and secondary mechanisms as potential therapeutic targets.

Vroman et al. comprehensively review generated animal models resembling the Ehlers–Danlos syndromes (EDS), a group of HCTD mainly characterized by skin hyperextensibility, joint hypermobility and generalized tissue fragility. Most EDS types are caused by genetic defects that affect connective tissue biosynthesis, thereby compromising collagen biosynthesis or fibrillogenesis and resulting in a disorganized extracellular matrix. For many of the EDS types animal models are available, providing a key advancement for studying the pathomechanisms underlying EDS and for developing new therapeutic avenues for EDS in preclinical trials.

Cutis laxa is a general term for a group of disorders that are characterized by lax, wrinkled skin that is sagging and lacks elasticity. Known causative mutations were reported in *ELN*, *FBLN4*, *LTBP4*, as well as in *ATP6V1E*1 or *ATP6V1A*, which encode the E1 and A subunits, respectively, of the V_1_ domain of the heteromultimeric V-ATPase complex. Mutations affecting the B1 (ATP6V1B1 and B2 (ATP6V1B2) subunits on the other hand cause autosomal recessive distal renal tubular acidosis with early-onset hearing loss and Zimmermann-Laband syndrome, respectively. Findings of Li et al. expand the variant spectrum of hearing-loss-associated genes and provide new insights on understanding of hearing-loss candidate genes *ATP6V1B2*, but also *TJP2*, and *KIF11*.

Fibrillin microfibrils represent the extracellular scaffolds onto which tropoelastin deposition occurs, followed by lysyl oxidase mediated crosslinking required for proper elastic fiber formation. Thereby, the targeting of tropoelastin is facilitated by adaptor proteins such as latent TGF-β binding protein-4 (encoded by *LTBP4*) which, when mutated leads to autosomal recessive cutis laxa type C (ARCL1C). Alanazi et al. show, using biochemical and biophysical approaches, that *LTBP4* mutations contribute to ARCL1C by disrupting the structure and interactions of LTBP-4 which are essential for elastogenesis in a range of mammalian connective tissues.

Mutations in fibrillin often lead to opposing clinical features ranging from tall to short stature, hyperflexible to stiff joints, or hyperextensible to stiff and fibrotic skin. The manuscript by Arnaud et al. features fibrillinopathies that are characterized by short extremities, and joint limitation described, as opposed the clinical hallmarks of Marfan syndrome (MFS). They provide an overview of the human genetic data and generated knockout mouse models targeting the involved genes, pointing towards a potential biological cooperation between A disintegrin and metalloprotease with thrombospondin-1-like domains (ADAMTS) enzymes or ADAMTS-like adaptor proteins and fibrillin-1 in the development of each other opposing clinical phenotypes.

Finally Mizumoto and Yamada summarize glycobiological aspects of congenital disorders caused by defects in glycosaminoglycan-biosynthetic enzymes including specific glysocyltransferases, epimerases, and sulfotransferases, in addition to core proteins of proteoglycans.

Overall, this Research Topic issue provides a broad picture of the intricate molecular pathways underlying HCTD which serve as model diseases to better understand how fundamental the extracellular microenvironment regulates cellular behavior.
